# Patterns of airway obstruction of non-acquired origin in children with and without major congenital anomalies

**DOI:** 10.1007/s00431-021-04198-6

**Published:** 2021-07-21

**Authors:** Rodrigo Gonçalves Dias, Roland Giger, Philipp Latzin, Thomas Riva, Carmen Casaulta, Francis Ulmer, Yves Jaquet, Lluís Nisa

**Affiliations:** 1 Department of Otorhinolaryngology Head and Neck Surgery Inselspital, Bern University Hospital, University of Bern, 3010 Bern, Switzerland; 2 Division of Respiratory Medicine, Department of Paediatrics, Inselspital, University of Bern, 3010 Bern, Switzerland; 3 Department of Anaesthesiology and Pain Therapy, Inselspital, Bern University Hospital, University of Bern, 3010 Bern, Switzerland; 4 Division of Respiratory Medicine, Department of Paediatrics, Inselspital, University of Bern, 3010 Bern, Switzerland; 5 Department of Paediatrics, Section of Paediatric Critical Care, Bern University Hospital, University of Bern, 3010 Bern, Switzerland; 6 Department of Otorhinolaryngology Head and Neck Surgery, Hôspital Neuchâtelois, 2000 Neuchâtel, Switzerland

**Keywords:** Airway obstruction, Congenital disorders, Airway endoscopy, Outcomes

## Abstract

Major congenital anomalies are known to play a role in the management and prognosis of airway obstruction. Most studies assess acquired forms of airway obstruction. Data on congenital or otherwise non-acquired forms of airway obstruction is sparse. In this retrospective, single-institution cohort study, we sought to evaluate and compare the patterns of airway obstruction in children with and without major congenital anomalies, and to assess the impact of management and outcome, irrespective of aetiology. Fifty-five patients were included, 23 with and 32 without underlying major congenital anomalies. Multilevel airway obstruction (usually affecting the nasopharynx, oropharynx, and the trachea) was more common in children with congenital anomalies (91% vs. 41%, *p* < .001). Consequently, these children required more frequent and earlier surgical management, especially tracheostomy and adenotonsillar surgery.

*Conclusions: *Major congenital anomalies are associated with multilevel airway obstruction and poor functional prognosis. A simple clinical definition considering impact of major congenital anomalies on development and growth may help guide management plans following endoscopic evaluation of the entire airway and flanked by multidisciplinary discussions.

**Table:** 

**What is Known:** *• Children with major comorbidities display increased disease severity and more prevalent multilevel airway obstruction* *• Previous studies include both children with acquired and non-acquired forms of airway obstruction; therefore, the actual impact major comorbidities in children with non-acquired causes of airway obstruction remain unclear.*	
**What is New:** *• A total of 42% children in this study population had major comorbidities with and impact on growth and/or psychomotor development, with a higher prevalence of multilevel airway obstruction and worse rates of functional improvement/recovery.* *• Children with major comorbidities require tracheostomy more often and earlier than those without major comorbidities, and remain tracheostomy-dependent for a longer time.*

**What is Known:**

*• Children with major comorbidities display increased disease severity and more prevalent multilevel airway obstruction*

*• Previous studies include both children with acquired and non-acquired forms of airway obstruction; therefore, the actual impact major comorbidities in children with non-acquired causes of airway obstruction remain unclear.*

**What is New:**

*• A total of 42% children in this study population had major comorbidities with and impact on growth and/or psychomotor development, with a higher prevalence of multilevel airway obstruction and worse rates of functional improvement/recovery.*

*• Children with major comorbidities require tracheostomy more often and earlier than those without major comorbidities, and remain tracheostomy-dependent for a longer time.*

## Introduction

Airway obstruction features reduced airflow through one or several airway levels, originating from an anatomical and/or functional size reduction of the airway lumen. When significant enough to create a turbulent airflow, airway obstruction usually manifests with respiratory noises (e.g. stridor, stertor, wheezing), as well as variable degrees of respiratory distress. From an etiological perspective, clinically useful classifications distinguish congenital from acquired, and acute from chronic airway obstruction.

The most common diagnoses in children with congenital upper airway obstruction include laryngomalacia, vocal cord paralysis, and subglottic stenosis, while subglottic haemangiomas, glottic webs, saccular cysts, and laryngotracheal clefts are less frequently encountered [1].

Formal airway assessment usually entails flexible and rigid endoscopic examination of the entire airway to characterize the site(s) and degree of obstruction [2–6]. Moreover, the presence of anomalies is a key element to consider in children with upper airway obstruction. For instance, neurological and cardiological comorbidities and synchronous airway lesions (especially subglottic stenosis and tracheomalacia) may be associated with less favourable outcomes following supraglottoplasty among patients with severe laryngomalacia [7, 8]. Similar observations apply to children with subglottic stenosis having undergone partial cricotracheal resection, in whom comorbidities are associated with delayed decannulation [9–11]. Underlying neurodevelopmental problems, prematurity, cardiac, and other congenital anomalies are in turn associated with multilevel airway lesions [12, 13]

Although it is generally accepted that children with underlying congenital anomalies tend to have more complex forms of upper airway obstruction, few studies have so far compared the patterns of obstruction in children with and without major congenital anomalies [12]. Moreover, most available studies include children with acquired lesions (mainly intubation-related), rendering the impact of these anomalies on obstruction patterns more difficult to predict. We thus aimed to assess the patterns of non-acquired forms of airway obstruction in treatment-naive children with and without major congenital anomalies, as well as the impact of such anomalies on management strategies and outcome.

## Methods

### Cohort, inclusion criteria, and data extraction

This study was approved by the Cantonal Ethical Committee of Bern. This retrospective study was conducted at a tertiary care centre. Potential patients for inclusion were identified through the prospectively documented computer-based records of the board for complex laryngotracheal pathologies in children and adults. This multidisciplinary airway board includes otolaryngologists, adult and paediatric pulmonologists, thoracic surgeons, and paediatric and adult intensive care specialists and anaesthetists. When needed, additional specialists are consulted.

We included patients between 0 and 16 years of age with an initial diagnosis of congenital or otherwise non-acquired airway obstruction (i.e. non-traumatic, non-infectious), treated between 1 Jan. 2015 and 31 Dec. 2018, for whom complete airway evaluation by means of diagnostic flexible endoscopy (spontaneous breathing) and direct pharyngolaryngoscopy had been performed. Only baseline, pre-therapeutic endoscopies were considered.

The data collected included gender, age, clinical presentation, presence and type of congenital anomalies, endoscopic findings (levels of obstruction), other diagnostic methods, management approaches, and outcome at last follow-up. Retrieved data were recorded in a standardized spreadsheet. Recorded data were double-checked in a non-blinded manner by two authors (RGD and LN). In case of discordance, agreement was reached through discussion.

### Endoscopic airway evaluation

All included children underwent complete endoscopic evaluation of the airway, including transnasal flexible endoscopy down to the bronchi following nasal decongestion and topical laryngeal anaesthesia with lidocaine 1%. For evaluation of spontaneous breathing under anaesthetic prior to any intubation, sevofluorane was used. Direct pharyngolaryngoscopy ± esophagoscopy were performed after administration of total intravenous anaesthesia.

### Outcome measures

Outcome following therapy at last follow-up was defined as *full recovery* in the absence of any clinical airway manifestation, *functional improvement* when children displayed physical abilities similar to peer groups of same age but with residual manifestations such as intermittent stridor or dysphagia, or minimally limited by exertional dyspnoea. *No improvement* described children exhibiting no significant changes in the initial clinical manifestations. With respect to children with tracheostomies, specific analyses were carried out separately as described in “Group comparisons and data analysis.” If children could be decannulated, we considered final outcome as either a full recovery or functional improvement, depending on residual clinical manifestations.

### Group comparisons and data analysis

Children were divided in two groups according to the presence or absence of major congenital anomalies, following criteria previously used by Ho-Wo-Cheong et al. and ourselves [12, 14]. The term “anomalies” is used instead of syndrome or sequence, given that unifying diagnosis can remain elusive in some children. The first group included patients with congenital anomalies impacting general health, emphasising severe psychomotor retardation and/or failure to thrive, usually within the context of a syndrome/sequence, or featuring major malformations of one or several organs. The second group consisted of children without major congenital anomalies. The latter group included children with a perinatal history pertinent for either minor or isolated organ malformations whose comorbidities did not affect their psychomotor development or growth.

After confirming normal distribution with the Shapiro Wilk test, we used Fisher’s exact test and Student’s t test to compare proportions and means. Time-dependent endpoints (age at time of tracheotomy and time of tracheostomy dependence) were analysed by plotting Kaplan-Meier curves and log-rank tests.

All p-values are two-sided, and *p* < .05 was deemed to indicate statistical significance.

## Results

### Demographics, clinical presentation, diagnostics, and comorbidities

A total of 55 children fulfilled the inclusion criteria, including 36 males and 19 females. Group 1 (major congenital anomalies) included 23 children (42%) and group 2 (children without major congenital anomalies or with minor/isolated co-morbidities) included 32 children (58%). The median age at initial presentation was 2 months (range 0–17 years). The most prevalent first manifestations at presentation were stridor (36%), feeding impairment or swallowing disorders/aspiration (33%), sleep-disordered breathing (23%), increased work of breathing (22%), and other nonspecific breathing sounds (18%). The children with sleep-disordered breathing included in this study underwent endoscopic evaluation due to severity of their clinical presentation not being sufficiently explained by their physical examination. Relevant diagnostic imaging studies of the chest, the head and the neck were performed when clinically indicated.

All children underwent diagnostic endoscopy. Table 1 lists the levels at which an airway obstruction was found during airway assessment, as well as synchronous lesions deemed to contribute to airway obstruction. Table 2 lists all associated non-airway conditions diagnosed in this cohort of children, as well as the specific diagnoses in the group of children with major congenital anomalies.

**Table 1:** Patterns, levels, and causes of airway obstruction in children with and without major congenital anomalies

**Characteristics**			Major congenital anomalies*		*p*-value
			Present (*n* = 23)	Absent (*n* = 32)	
Gender		Male	14	22	.577
		Female	9	10	
Age at first manifestation (median, range, months)**			0.17 (0–8.7)	0.19 (0–16.76)	.356
Level of obstruction**, n (%)		Single	2 (8.7%)	19 (59%)	<.001
		Multiple	21 (91%)	13 (41%)	
**Site of obstruction**	Specific pathologies, *n*				
*Nasal cavity* *n* = 4 (7.3%)	Choanal stenosis = 1; turbinate hyperplasia = 2; stenosis of piriform aperture = 1		2 (8.7%)	2 (6.2%)	1.000
*Nasopharynx* *n* = 21 (38%)	Obstructive adenoid hypertrophy = 21		14 (61%)	7 (22%)	.005
*Oropharynx* *n* = 30 (55%)	Tonsils hypertrophy = 17		13(57%)	4(12%)	.001
	Base of tongue hyperplasia = 13		11 (48%)	2 (6.3%)	.001
*Supraglottic larynx* *n* = 22 (40%)	Laryngomalacia = 14; others = 8		11 (48%)	11 (34%)	.406
*Glottic larynx* *n* = 12 (22%)	Vocal cord palsy = 5; paradoxical vocal cord dysfunction/dyskinesia = 1; vocal cord cyst = 1; stenosis = 3; glottis hamartoma = 1; others = 2†		4 (17%)	8 (25%)	.742
*Subglottic larynx* *n* = 13 (24%)	Subglottic stenosis = 9; subglottic cyst = 2; subglottic haemangioma = 1; others = 1		4 (17%)	9 (28%)	.522
*Trachea* *n* = 15 (27%)	Stenosis = 5; congenital tracheo-esophageal fistula = 2; tracheal narrowing due to external compression = 1		5 (22%)	3 (9.4%)	.257
	Tracheomalacia = 7		6 (26%)	1 (3.1%)	.017
*Bronchi/lungs* *n* = 13 (24%)	Lung/bronchial malformation = 8		4 (17%)	3 (9.4%)	.435
	Bronchomalacia = 5		4 (17%)	1 (3.1%)	.146

^*^Other than congenital/non-acquired airway obstruction

^**^Multiple anatomical levels possible

^†^One patient in this group had more than one cause of glottic obstruction

**Table 2:** Associated non-airway conditions and major congenital anomalies*

Associated conditions (n = 55)		Overall (*n*, %)	Major anomalies (*n*)	
			Present	Absent
*Type of comorbidities*	Cardiologic	14 (25)	11	3
	Neurologic	11 (20)	11	0
	Psychomotor retardation	13 (24)	13	0
*Prematurity*		13 (24)	8	5
*Craniofacial malformations*	Retrognathia a/o micrognathia	4 (7.3)	4	0
	Mandibular dysplasia	1 (1.8)	1	0
	Craniosynostosis	1 (1.8)	1	0
	Relative macrocephaly	1 (1.8)	1	0
	Trigonocephaly	2 (3.6)	2	0
	Hydrocephalus internus	1 (1.8)	1	0
	Microcephaly	1 (1.8)	1	0
	Undetermined (rough facial features)	1 (1.8)	1	0
*Sleep apnoea*		7 (13)	4	3
*Swallowing disorder*		17 (31)	13	4
*Gastrointestinal comorbidities*	Gastroesophageal reflux disease	6 (11)	2	4
	Other	6 (11)	5	1
Major congenital anomalies (*n* = 23)		Patients (%)		
Trisomy 21		4 (17)		
Brain malformation		2 (8.7)		
Undefined dysmorphic association/syndrome		5 (22)		
Pfeiffer syndrome		1 (4.3)		
Trisomy 1q und deletion 5p		1 (4.3)		
Duchenne muscular dystrophy		1 (4.3)		
Deletion 1q22		1 (4.3)		
Caudal regression syndrome		1 (4.3)		
Goldenhar syndrome		1 (4.3)		
Microdeletion 22q11		1 (4.3)		
Feingold-syndrome		1 (4.3)		
Pallister-Hall-syndrome		1 (4.3)		
Deletion chromosome 4 q32.3/q35.2, duplication chromosome 10 p15.3/p15.1 and Fallot tetralogy		1 (4.3)		
Combined cardiac and pulmonary malformation		1 (4.3)		
Deletion in exon 4 of the 3-beta-HSD II gene		1 (4.3)		

^*^More than one condition possible

### Patterns of airway obstruction in children with and without major congenital anomalies

Gender distribution and median age at diagnosis were not significantly different between these groups (Table 1).

With respect to obstruction patterns (Table 1), children with major congenital anomalies showed a significantly higher prevalence for multilevel airway obstruction when compared to children without major congenital anomalies (91% vs. 41%, *p* < .001). Regarding specific sites of obstruction, the nasopharynx (*p* = .005), the tonsils (*p* = .001), the base of the tongue (*p* = .001), and the trachea in the presence of tracheomalacia (*p* = .017) were significantly more frequent sites of obstruction in children with major congenital anomalies than in children without (Table 1). None of the sites of obstruction were more prevalent in children without major anomalies.

### Treatment and outcome

**Fig. 1:**
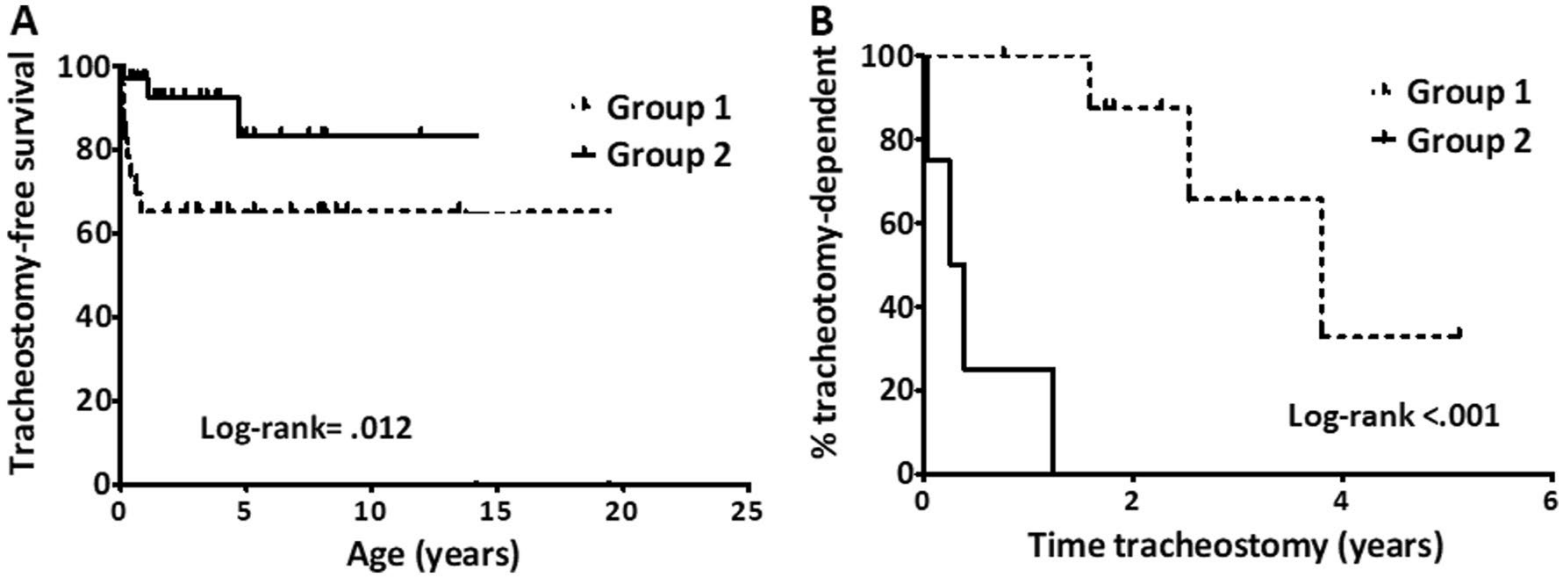
Kaplan-Meier representation of tracheostomy likelihood (panel **A**) and tracheostomy dependence time (panel **B**) in children with (Group 1) and without (Group 2) major congenital anomalies

Seventy-six percent (*n* = 42) of all children underwent at least one surgical procedure (Table 3). The remaining patients (*n* = 13) were treated conservatively. The vast majority of children with major anomalies (91%) and approximately two-thirds of children without major anomalies (66%) required surgery, yielding a difference that was not significant (*p* = .051). Tracheostomy (*p* = .028) and adenotonsillectomy (*p* = .018) were more commonly performed in children with major congenital anomalies. No other procedures were carried out more commonly in either group (Table 3). With respect to tracheostomy, children with major congenital anomalies were significantly younger at the time of tracheostomy than those without (14.20 vs. 19.47 months, respectively, *p* = .012; Fig. 1A).

**Table 3:** Treatment and outcome

Features		Major congenital anomalies		*p*-value
		Present (%)	Absent (%)	
Type of surgery				
*Airway surgery (any approach)*		21 (91)	21 (66)	.051
*Tracheotomy*		9 (39)	4 (12)	.028
*Other tracheal surgery*		3 (13)	3 (9.4)	.686
*Adenotonsillar surgery*		12 (52)	6 (19)	.018
*Laryngeal surgery*		6 (26)	14 (44)	.257
Treatment outcome				
Airway symptoms*	Full recovery or improvement	16 (73)	29 (91)	.136
	No improvement	6 (27)	3 (9)	

^*^One child was lost to follow-up

Relative to outcome, full recovery or improvement of airway symptoms was seen in 82% (*n* = 45) of children at last follow-up, with no improvement in the remaining 16% (*n* = 9). One child in the group with major congenital anomalies was lost to follow-up. In terms of functional outcome, 73% of children with congenital anomalies and 91% without showed full recovery or improvement (*p* = .136).

Finally, all children without congenital anomalies that underwent tracheostomy were decannulated within a time frame of no more than 1.22 years. By contrast, 6/9 (67%) children with congenital anomalies were still tracheostomy-dependent at last follow-up (Fig. 1B).

## Discussion

This study compares the patterns of congenital or otherwise non-acquired airway obstruction and its impact on management and outcome among children with and without major congenital anomalies. The main findings include the following: (1) 91% of children with major congenital anomalies and 41% without presented multilevel airway obstruction, (2) children with major congenital anomalies require tracheostomy more frequently and earlier in life than children without, and (3) children with major congenital anomalies remain tracheostomy-dependent for a longer period than children without (irrespective of the aetiology).

The presence of congenital anomalies has been shown to influence outcome and clinical course of several airway conditions, including laryngomalacia, vocal cord paralysis, and subglottic stenosis [7–9, 13–23].

In contrast to previous studies, in this study, we excluded acquired causes of airway obstruction. In total, 62% of patients presented with multilevel obstruction, with a prevalence of 91% in the group with major anomalies and 41% in the group with minor anomalies. As other studies assessing the presence of synchronous airway lesions included patients with intubation-related injuries, we cannot directly compare the prevalence of multilevel obstruction [12, 19, 24]. Prematurity has been shown to be associated with multilevel upper airway obstruction, most commonly affecting the larynx. Such lesions are usually related to prolonged intubation, irrespective of associated comorbidities [24].

Existing studies tend to focus exclusively on the laryngotracheal airway when assessing for upper airway lesions and fail to examine the nasal, nasopharyngeal, oropharyngeal, tracheal, and bronchial segments of the airway. It is imperative to consider adenotonsillar pathology in the paediatric population, particularly among children with major congenital anomalies, as this pathology represents a common source of upper airway obstruction. This is especially important in the context of obstructive sleep apnoea syndrome, necessitating an endoscopic evaluation to systematically assess the static and dynamic aspect of the entire airway, covering all areas of potential airway obstruction from the nostrils down to the bronchi [25–28].

An important aspect of relying on purely clinical criteria is that a final diagnosis does not need to be reached to assess the likely patterns of airway obstruction and outcome.

As shown in previous studies, this cohort confirmed that children with major congenital anomalies are more likely to require a tracheostomy [8, 9, 14]. This is possibly related to the increased occurrence of multilevel obstruction. Moreover, at our institution, patients with major congenital anomalies underwent adenotonsillar surgery frequently. This could be explained by the fact that prior to tracheostomy removal, children undergo a comprehensive diagnostic endoscopy to re-evaluate the airway. Surgical closure of the tracheostomy is usually scheduled for a later time. In children, presenting obvious adenoid and/or tonsillar obstruction, we tend to perform adenotonsillotomy prior to tracheostomy removal. Our current results do not allow elaborate on the prognostic impact of performing adenotonsillar surgery prior to decannulation.

The presence of major congenital anomalies was linked to a longer tracheostomy dependence. The information obtained in this study will allow healthcare providers to tailor their management approaches to the needs of the individual patients and provide patients and parents with more accurate information relative to outcomes. Another implication is the understanding that airway surgery is likely to be less effective in children with major congenital anomalies, and that in the event of significant airway compromise a tracheostomy may be the best primary treatment to relieve obstruction.

Several limitations need to be acknowledged in this study, first, the limitations inherent to its retrospective design, which could be partially mitigated by the systematic collection of data within the frame of our airway board. Second, endoscopic findings are subject to interobserver bias. While a prospective evaluation of images would have enabled to review the images in a blinded manner to address such bias, all diagnostic endoscopies conducted among children included in this study were performed in presence of at least one senior paediatric respiratory medicine physician and a senior otolaryngologist. Finally, a validated tool to evaluate outcomes, such as repeat airway assessment, polysomnography, or when feasible, pulmonary function tests would certainly have added value to the current results. Such measurements need to be performed in upcoming prospective studies.

## Conclusion

Children with presence of major congenital anomalies have a significantly higher prevalence of multilevel airway obstruction. These children require tracheostomy earlier and tend to remain tracheostomy-dependent for a longer time. Recognizing this may help tailoring therapeutic strategies to the individual needs of the patient and facilitates informing parents regarding anticipated outcomes.

## Data Availability

Available on request.
