# Correction to: Patterns of airway obstruction of non-acquired origin in children with and without major congenital anomalies

**DOI:** 10.1007/s00431-024-05644-x

**Published:** 2024-08-09

**Authors:** Rodrigo Gonçalves Dias, Roland Giger, Philipp Latzin, Thomas Riva, Carmen Casaulta, Francis Ulmer, Yves Jaquet, Lluís Nisa

**Affiliations:** 1 Department of Otorhinolaryngology Head and Neck Surgery Inselspital, Bern University Hospital, University of Bern, 3010 Bern, Switzerland; 2 Division of Respiratory Medicine, Department of Paediatrics, Inselspital, University of Bern, 3010 Bern, Switzerland; 3 Department of Anaesthesiology and Pain Therapy, Inselspital, Bern University Hospital, University of Bern, 3010 Bern, Switzerland; 4 Department of Paediatrics, Section of Paediatric Critical Care, Bern University Hospital, University of Bern, 3010 Bern, Switzerland; 5 Department of Otorhinolaryngology Head and Neck Surgery, Hôspital Neuchâtelois, 2000 Neuchâtel, Switzerland

**Correction to: European Journal of Pediatrics (2021) 181:303–309**

10.1007/s00431-021-04198-6

During the typesetting of the published article entitled “Patterns of airway obstruction of non acquired origin in children with and without major congenital anomalies”, the co-last authorship statement of authors Yves Jaquet and luís Nisa is not mentioned.

The original article has been corrected.

